# Distinct energetics and closing pathways for DNA polymerase β with 8-oxoG template and different incoming nucleotides

**DOI:** 10.1186/1472-6807-7-7

**Published:** 2007-02-21

**Authors:** Yanli Wang, Tamar Schlick

**Affiliations:** 1 Department of Chemistry and Courant Institute of Mathematical Sciences, New York University, 251 Mercer Street, New York, NY 10012, USA

## Abstract

**Background:**

8-Oxoguanine (8-oxo**G**) is a common oxidative lesion frequently encountered by DNA polymerases such as the repair enzyme DNA polymerase β (pol β). To interpret in atomic and energetic detail how pol β processes 8-oxo**G**, we apply transition path sampling to delineate closing pathways of pol β 8-oxo**G **complexes with d**C**TP and d**A**TP incoming nucleotides and compare the results to those of the nonlesioned **G**:d**C**TP and **G**:d**A**TPanalogues.

**Results:**

Our analyses show that the closing pathways of the 8-oxo**G **complexes are different from one another and from the nonlesioned analogues in terms of the individual transition states along each pathway, associated energies, and the stability of each pathway's closed state relative to the corresponding open state. In particular, the closed-to-open state stability difference in each system establishes a hierarchy of stability (from high to low) as **G**:**C **> 8-oxo**G**:**C **> 8-oxo**G**:**A **> **G**:**A**, corresponding to -3, -2, 2, 9 k_B_T, respectively. This hierarchy of closed state stability parallels the experimentally observed processing efficiencies for the four pairs. Network models based on the calculated rate constants in each pathway indicate that the closed species are more populated than the open species for 8-oxo**G**:d**C**TP, whereas the opposite is true for 8-oxo**G**:d**A**TP.

**Conclusion:**

These results suggest that the lower insertion efficiency (larger K_m_) for d**A**TP compared to d**C**TP opposite 8-oxo**G **is caused by a less stable closed-form of pol β, destabilized by unfavorable interactions between Tyr271 and the mispair. This stability of the closed vs. open form can also explain the higher insertion efficiency for 8-oxo**G**:d**A**TP compared to the nonlesioned **G**:d**A**TP pair, which also has a higher overall conformational barrier. Our study offers atomic details of the complexes at different states, in addition to helping interpret the different insertion efficiencies of d**A**TP and d**C**TP opposite 8-oxo**G **and **G**.

## Background

DNA polymerases are vital enzymes that assist DNA replication and maintain the genomic stability of living organisms. They are grouped into seven main families: A, B, C, D, X, Y, and RT. While DNA polymerases from the A, B, C, and D families are replicative, others from the X and Y families repair (or bypass) DNA damage or replication errors. Oxidative DNA damage, arising from modification of DNA by oxygen-derived species, is a frequent type of damaged DNA in cells that utilize oxygen [1,2]. If not accurately repaired, oxidative DNA damage can ultimately lead to various human diseases, such as various cancers and premature aging [3-7]. Thus, understanding the mechanism by which DNA polymerases process lesions is an important biological challenge.

**Figure 1:**
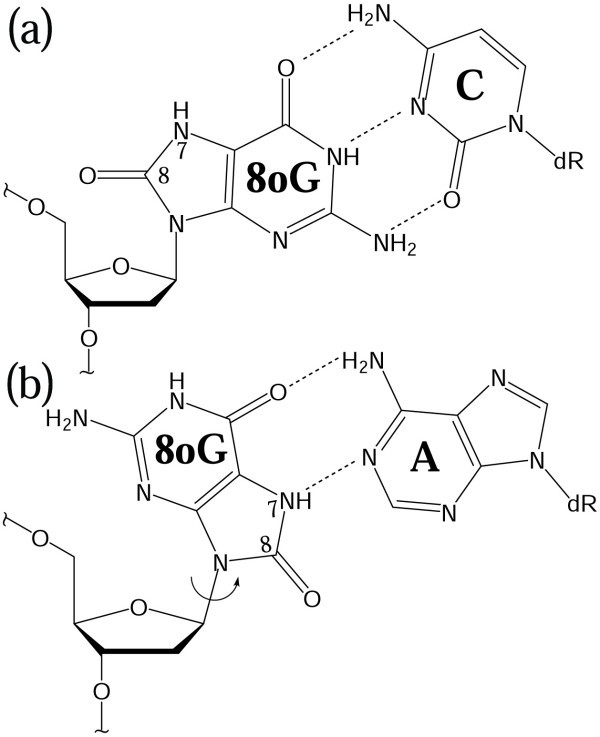
**The base pairing possibilities of 8-oxoG (8oG)**. In an *anti *conformation it forms a Watson-Crick base pair with d**C**TP (a); by assuming a *syn *conformation, it can form a Hoogsteen base pair with d**A**TP.

One of the most common oxidative lesions in the genome is 7, 8-dihydro-8-oxoguanine, also called 8-oxoguanine or 8-oxo**G **(Fig. 1). Due to oxidation of C8 to a carbonyl group, the resulting 8-oxo**G **can adopt a stable *syn *conformation by rotating 180° around the glycosidic bond when pairing with d**A**, in addition to the regular *anti *conformation when pairing with d**C**. Since the 8-oxo**G**:d**A **mispair can introduce **G **to **T **transversions, enzymes such as MutY in bacteria and OGG1 and MYH in eukaryotes exist to repair/excise the incorrect bases [8-10]. A recent crystal structure of MutY complexed with 8-oxo**G **[11] reveals the extensive array of hydrogen bonds that allow MutY to break the link between such a mispaired adenine (i.e., linked to 8-oxo**G**) while not affecting correctly paired **A**:**T **or **G**:**C **units.

The 8-oxo**G **type of DNA damage is frequently encountered by DNA polymerases, including the repair enzyme DNA polymerase β (pol β). Kinetic studies [12] have shown that pol β prefers correct insertion (d**C**TP) to incorrect insertion (d**A**TP) opposite an 8-oxo**G **template. As in pol β, d**C**TP is also more likely to be incorporated opposite 8-oxo**G **in many other DNA polymerases, such as RB69, Dpo4, and wild-type T7 [13-15]. Furthermore, although d**A**TP is less favorably inserted compared to d**C**TP opposite 8-oxo**G **in pol β, the efficiency of d**A**TP incorporation opposite 8-oxo**G **is approximately 62,000-fold higher than opposite d**G **[12]. In other words, the 8-oxo**G**:d**A**TP base pair appears to be treated by pol β like a regular Watson-Crick base pair (**G**:**C**) rather than a **G**:**A **mispair.

Crystallographic structures of various DNA polymerase complexes including RB69, T7, *Bacillus *fragment (BF), and Dpo4 with 8-oxo**G **as the template base at the DNA primer/template junction have been resolved [13-18]. In most structures, the 8-oxo**G **template pairs with d**C**TP in an *anti*:*anti *fashion by kinking the template backbone to avoid the steric clash between O8 and O5' atoms of 8-oxo**G**. However, few crystal structures of DNA polymerases have captured 8-oxo**G **(*syn*) pairing with a d**A**TP incoming nucleotide. By mutating an active-site residue (Lys536) to alanine, Brieba et al. have successfully crystallized the ternary complex of T7 DNA polymerase with 8-oxo**G **(*syn*):d**A**TP [16]. In the Dpo4/DNA ternary structure [18], 8-oxo**G **assumes a *syn *conformation pairing with d**A**TP but a second d**A**TP nucleotide is also found at the active site dissecting the minor groove. In the crystal structure of pol β bound to DNA with 8-oxo**G **[19], the enzyme is in an open conformation and the adenine base of d**A**TP stacks with the template base, both assuming *anti *conformations.

Molecular dynamics simulations of pol β/DNA substrate complexes with 8-oxo**G **[20] have shown that the closing conformational pathway of pol β is slowed by d**A**TP insertion opposite 8-oxo**G **(the thumb subdomain cannot attain a fully closed conformation after 18 ns simulation) but that the closing process is unaffected by d**C**TP opposite 8-oxo**G**. Specifically, unfavorable interactions between the nascent base pair and Tyr271 on the thumb subdomain cause the adenine base on d**A**TP to flip to a *syn *conformation when 8-oxo**G**:d**A**TP is bound to the pol β/DNA substrate complex.

To delineate the factors that cause different efficiencies for d**A**TP and d**C**TP insertion opposite 8-oxo**G **as well as opposite d**G**, we apply transition path sampling (TPS) simulations to further dissect the closing pathways of pol β associated with 8-oxo**G **and different incoming nucleotides (d**A**TP and d**C**TP). We only consider the 8-oxo**G **(*anti*):d**C**TP (*anti*) (abbreviated as 8-oxo**G**:d**C**TP) and 8-oxo**G **(*syn*):d**A**TP (*anti*) (8-oxo**G**:d**A**TP) conformations here because these two are usually found in a DNA duplex. Earlier dynamics simulations also indicate that the *anti*:*anti *conformation is less favorable than the *syn*:*anti *conformation for the 8-oxo**G**:d**A**TP mispair in pol β [20].

The TPS method developed by Chandler and co-workers [21,22] can traverse high barriers on the potential energy surface and capture rare events that are not accessible to classical simulations. TPS has been applied to study various systems ranging from clusters of small molecules such as ions, water, peptides and lipids [23-30] to large complex systems [31,32]. Recently, TPS was successfully implemented to study the closing pathways of pol β complex binding **G**:**C **and **G**:**A **base pairs at the active site [31,32]. The free energy profiles of the **G**:**C **and **G**:**A **complexes help explain the different efficiencies of the correct and incorrect incorporations by pol β; essentially, a higher overall closing barrier for the mispair, multiple closing pathways in the mispair, and instability of the closed **G**:**A **system were identified as crucial factors to explain fidelity, discrimination in pol β's repair cycle. Our present work aims to resolve the complete conformational pathways of pol β associated with an oxidative lesion 8-oxo**G **to advance our understanding of the fidelity mechanism of pol β in processing a lesion. Although we expected energy differences to be small, systematic differences can help interpret biological observations.

In addition, to confirm the role of Tyr271 in destabilizing the closed conformation of the pol β/DNA/8-oxo**G **(*syn*):d**A**TP complex, we perform an MD simulation for a Y271A mutant of this lesioned mismatch complex before chemistry. With the bulkier Tyr271 replaced by alanine, the thumb subdomain of pol β is expected to close faster onto the nascent base pair than in the wild-type simulation [20].

## Results

### Conformational events during thumb closing differ in the 8-OxoG:dATP and 8-OxoG:dCTP complexes

By performing TMD simulations on the two 8-oxo**G **complexes, we obtained two initial trajectories that link their open and closed states. The time courses of side-chain dihedral angle motions and the RMSD of α-helix N are shown in [Additional file 1] (see additional figures 1 and 2). From these plots, we initially identified tentative transition states (Table 1) for the two pol β 8-oxo**G **complexes. The existence of these transition states was further confirmed by unconstrained simulations on configurations selected from the TMD trajectory [see Additional file 1] (see additional figures 3 and 4).

As shown by Table 1, both pol β 8-oxo**G **complexes have 4 transition states, but variances in the ranges of the associated order parameters occur. For both complexes, the first and fourth transition states are the same – thumb subdomain closing and Phe272 flip, respectively. For the 8-oxo**G**:d**A**TP system, the second and third transition states are Asp192 flip and Arg258 rotation, while for the 8-oxo**G**:d**C**TP system, transition states 2 and 3 consist of Arg258 half-rotation and Arg258 full-rotation. The completion of the Arg258 rotation in two steps resembles the reference nonlesioned **G**:**C **complex [31]. However, unlike the subsequent rotation of a single dihedral angle (Cγ-Cδ-Nε-Cζ) of Arg258 in **G**:**C**, Arg258 rotation for 8-oxo**G**:d**C**TP involves conformational changes of the dihedral angle Cβ-Cγ-Cδ-Nε from -95° to -150° followed by Cγ-Cδ-Nε-Cζ from 100° to 150°. Moreover, the Phe272 flip (from -70° to -150°) in the 8-oxo**G**:d**C**TP system occurs through a direction opposite to that found for the nonlesioned **G**:**C**, where it changes from -70° to 40°.

Starting from the physical trajectories linking the open and closed states of the key residues and the thumb subdomain, we pieced the trajectory space of each transition by TPS simulations. This sampling involves generating trajectories connecting the initial and final states of each transition using shooting and shifting algorithms [33] and a Monte Carlo in the trajectory space [34]. For each transition region we collected 100 successful trajectories connecting the initial and final states. The adequate length of the sampling trajectories is ensured by examining their correlation functions [see Additional file 1] (see additional figures 5 and 6).

**Figure 2:**
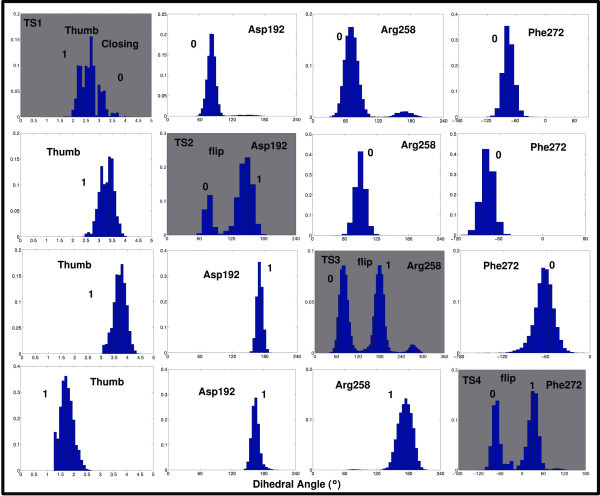
**Order parameter distributions in the 8-oxoG:dATP system**. Normalized probability distributions of the order parameters of transition states 1 to 4 (TS1 to TS4) in the 8-oxo**G**:d**A**TP complex. Labels 0 and 1 indicate the open and closed state conformations of the residues and thumb subdomain.

Figure 2 shows the probability distribution of the order parameters in transition states 1 to 4 of the 8-oxo**G**:d**A**TP complex. The existence of two peaks (marked by 0 and 1) on one plot implies a conformational transition across the energy barrier. Thus, a path sampling trajectory of TS1 to TS4 (pieced from hundreds of short continuous trajectories) captures the entire conformational pathway.

As seen from Fig. 2, when the thumb closes (TS1), all other residues are still in open states; when Asp192 flips (TS2), Arg258 and Phe272 are still unflipped (marked by 0); similarly, Arg258 rotates in TS3 before Phe272 flips in TS4.

During the transitions of Asp192 and Arg258 in the 8-oxo**G**:d**A**TP complex, the RMSD values of α-helix N relative to the closed structure are 2.5–4.0 Å and 3.0–4.2 Å, respectively, and the thumb fully closes prior to TS4 (RMSD of α-helix N is around 1.2–2.5 Å).

**Figure 3:**
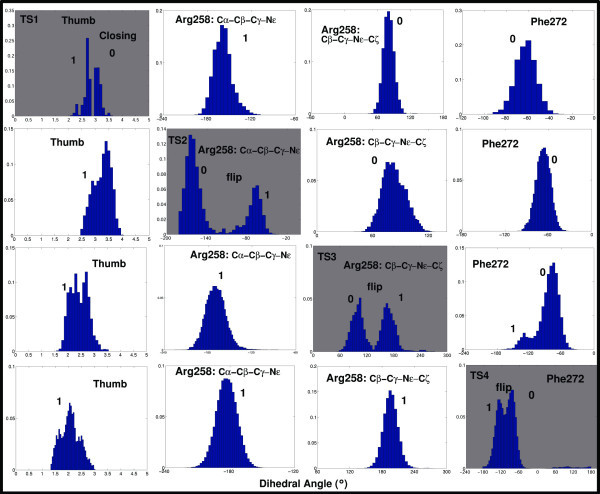
**Order parameter distributions in the 8-oxoG:dCTP system**. Normalized probability distributions of the order parameters in TS1 to TS4 in the 8-oxo**G**:d**C**TP complex. Labels 0 and 1 indicate the open and closed states of the residues and thumb subdomain.

Figure 3 shows normalized probability distributions of order parameters for the 8-oxo**G**:d**C**TP complex. During the Arg258 half-rotation in TS2, the second Arg258 order parameter (Cβ-Cγ-Nε-Cζ) and Phe272 still assume the open conformation; following TS3, Arg258 (Cβ-Cγ-Nε-Cζ) rotates completely. Finally, when Phe272 flips (TS4), all residues and the thumb subdomain are in the closed states.

Examining the RMSD of α-helix N (relative to the closed structure) during each transition in the 8-oxo**G**:d**C**TP complex, we determine that the values for TS2 to TS4 are 2.5–4.0 Å, 1.6–3.6Å, and 1.3–2.9 Å, respectively, smaller than their counterparts in the 8-oxo**G**:d**A**TP system. This suggests that the thumb subdomain closes faster relative to the side-chain motions in the 8-oxo**G**:d**C**TP complex than thumb closing does in the 8-oxo**G**:d**A**TP complex. Indeed, MD simulations [20] also indicate that the pol β complex approaches the closed state more quickly when it binds the 8-oxo**G**:d**C**TP base pair than the 8-oxo**G**:d**A**TP mispair.

### Free energy profiles of Pol β 8-OxoG complexes suggest different stability of their closed states

**Figure 4:**
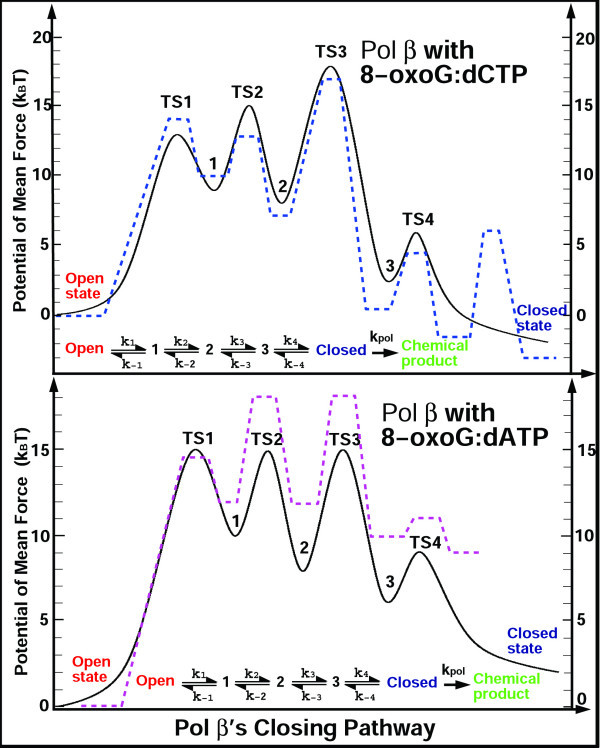
**The resolved free energy profiles**. The free energy profiles of the 8-oxo**G**:d**C**TP and 8-oxo**G**:d**A**TP complexes during pol β's thumb closing. The superimposed dashed lines are approximate free energy profiles of nonlesioned **G**:**C **(blue) and **G**:**A **(magenta) systems [32], respectively.

The free energy barriers (Table 1) for the transition states are estimated using our umbrella sampling protocol "BOLAS" [34] tailored for TPS. The energetic landscapes of the matched and mismatched 8-oxo**G **complexes in Figure 4 exhibit different characteristics. Although each system crosses four transition states for closing, the 8-oxo**G**:d**C**TP system must overcome a higher overall conformational barrier arising from TS3, namely the Arg258 rotation (18 ± 3 k_B_T), while the 8-oxo**G**:d**A**TP complex passes three energy barriers (TS1 to TS3) with similar magnitudes (15 ± 3 k_B_T). Hence, the rate-limiting conformational step in the matched system is Arg258 rotation, and that in the mismatch can be identified as thumb subdomain closing, since the latter requires the longest time (see τ values in Table 1) to cross the same energy barrier compared to Asp192 flip and Arg258 rotation. Specifically, the free energy barrier for thumb motions in the mismatched system is higher by 2 k_B_T than that in the matched system, but, because of the size of the error bar, we cannot determine if this finding is significant.

Moreover, the closed state of the matched system (-2 ± 3 k_B_T) is more stable than the mismatched complex (2 ± 3 k_B_T) relative to the corresponding open states (Fig. 4). Compared with available data for **G**:**C **and **G**:**A **[32], the closed states of the 8-oxo**G **complexes are slightly less stable than or as stable as **G**:**C **(-3 ± 3 k_B_T) but much more stable than **G**:**A **(9 ± 3 k_B_T). Thus, the sequence of stability for the 8-oxo**G **and **G **closed complexes compared to the corresponding open states is: **G**:**C **> 8-oxo**G**:d**C**TP > 8-oxo**G**:**A **> **G**:**A**.

Based on these free energy values, we compute the rate constants for each transition (see detailed protocol in Ref. [32]) (Table 1) and perform stochastic dynamics simulations on the conformational pathways using the Gillespie algorithm [35] implemented in the STOCKS program as done previously [32,36]. The kinetic networks of the conformational and chemical pathways for the two systems are sketched in Fig. 4. The catalytic constants of pol β (k_cat_) in processing the 8-oxo**G **match and mismatched base pairs can be roughly estimated from the stochastic simulations. For both complexes, we use the same chemical barrier (27 k_B_T) as that of the regular **G**:**C **matched base pair [37], because the steady-state k_cat _values for d**A**TP and d**C**TP incorporation opposite 8-oxo**G **are close to that for d**C**TP opposite d**G **[12].

**Figure 5:**
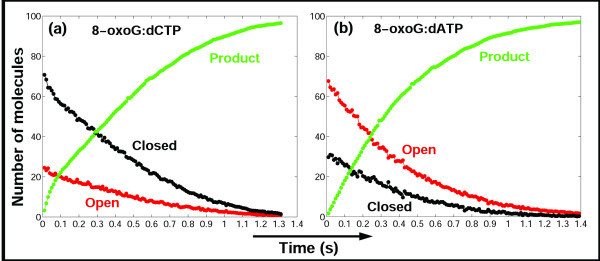
**Simulation results based on the network models**. The mean trajectories of the match (a) and mismatch (b) 8-oxo**G **systems obtained from 100 independent simulations with STOCKS [36]. The red, black, and green curves describe the evolution of the open, closed, and chemical product species, respectively.

The averaged time evolutions of the two systems are plotted in Figure 5, indicating how 100 complexes evolve in time. The majority (>60%) of the 100 starting open 8-oxo**G**:d**A**TP complexes converts to the chemical product in 0.4 s, corresponding to k_cat _= 2.5 s^-1^, while the same amount of product is produced for 8-oxo**G**:d**C**TP in 0.5 s (k_cat _= 2 s^-1^). Borrowing the experimental values of K_m _for d**C**TP and d**A**TP insertions (1.9 ± 0.36 and 6 ± 1.5 μM [12], respectively), we derive the catalytic efficiencies (k_cat_/K_m_) for correct and incorrect insertions as approximately 1.05 × 10^6 ^and 0.41 × 10^6 ^M^-1^·s^-1^, respectively. The ~2.5-fold more efficient d**C**TP insertion compared to d**A**TP opposite 8-oxo**G **agrees with the experimental data [12] that d**C**TP insertion is 2-fold more efficient.

### The role of active-site residue Tyr271

**Figure 6:**
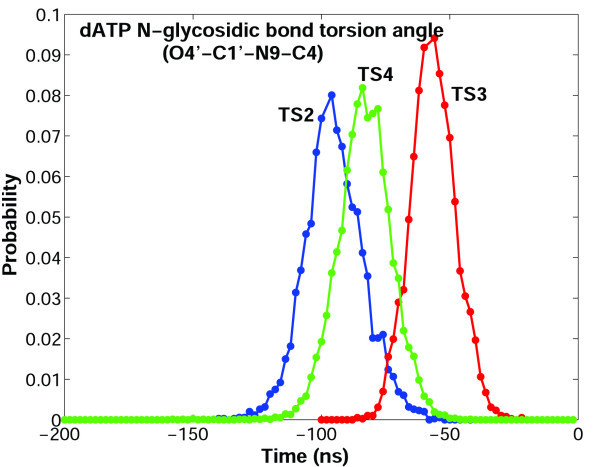
**Conformation of dATP in the 8-oxoG:dATP complex**. The probability distributions of the N-glycosidic bond torsion angle of d**A**TP during transition states 2 (Asp192 flip, blue), 3 (Arg258 rotation, red), and 4 (Phe272 flip, green) in the 8-oxo**G**:d**A**TP complex.

Our MD simulations [20] identified Tyr271 as a contributor to the thumb's slow closing in the 8-oxo**G**:d**A**TP complex, where we found that the incoming d**A**TP flips to a *syn *conformation due to interactions with Tyr271. Here our monitoring of the interactions between Tyr271 and the nascent base pair reveals that the binding of d**A**TP to the active site of pol β destabilizes the closed thumb conformation. Specifically, the probability distributions of the N-glycosidic bond torsion angle of d**A**TP during transition states 2 to 4 in the 8-oxo**G**:d**A**TP system (Fig. 6) show that d**A**TP changes from *anti *(TS2, -100°) to *syn *conformation (TS3, -56°) and returns to *anti *during TS4 (-86°). Interestingly, during TS3 (Arg258 rotation) the thumb subdomain appears more open (RMSD: 3.0–4.5 Å) than that in TS2 (RMSD: 2.5–4.0 Å). The repulsion between Tyr271 and d**A**TP thus slows thumb closing when Arg258 rotates by destabilizing the active site of pol β.

In contrast, transition path sampling simulations did not capture similar changes in the N-glycosidic dihedral angle of d**C**TP in the 8-oxo**G**:d**C**TP system throughout the sampling of all transition states.

**Figure 7:**
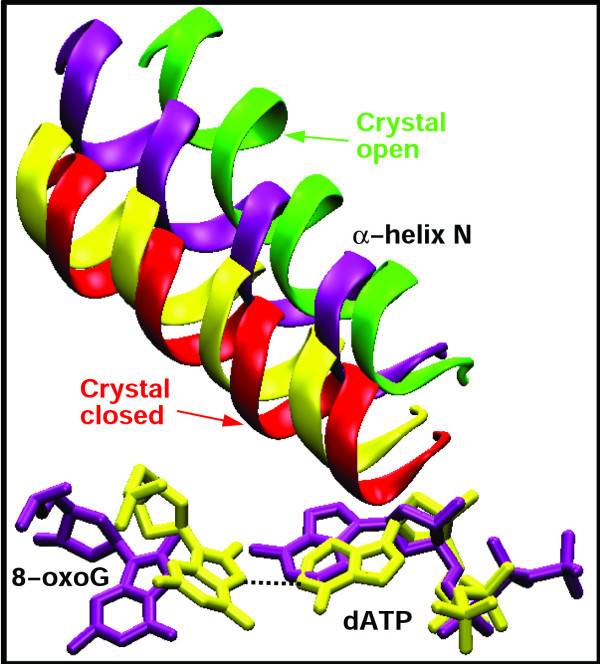
**The α-helix N and the nascent base pair conformations in the wild-type and mutant 8-oxoG:dATP complexes**. The final conformations of α-helices N and the nascent base pair in the wild-type (magenta) and Y271A mutant (yellow) pol β complex simulations with 8-oxo**G**:d**A**TP are superimposed according to their palm subdmains and compared to the crystal open (green) and closed (red) structures.

To further validate the role of Tyr271 in destabilizing the pol β 8-oxo**G **complex binding d**A**TP, we perform an MD simulation on the Y271A mutant of the mismatched system. With Tyr271 replaced by an alanine, the mutant complex closes quickly in the simulation of the intermediate state. The final conformations of α-helices N for both wild-type and mutant pol β complexes in Figure 7 (magenta vs. yellow) and corresponding RMSD plots of α-helices N of these complexes relative to the open and closed crystal structures [see Additional file 1] (see additional figure 9) also clarify the trends.

In the Tyr271Ala system, d**A**TP remains in the *anti *conformation and the thumb fully closes onto the nascent base pair. In addition, the conformations of the adenine bases in the wild-type (magenta) vs. mutant (yellow) complexes in Fig. 7 show that, unlike the distorted nascent base pair in the wild-type complex, the 8-oxo**G**:d**A**TP base pair in the mutant system becomes co-planar by adopting a Hoogsteen conformation (by the end of the simulation).

Thus, the adenine base flipping when d**A**TP is opposite 8-oxo**G**, as revealed from the TPS and MD simulations, and the stable d**A**TP conformation when Tyr271 is mutated to Ala, confirm that Tyr271 provides pol β with subtle selectivity for distinguishing d**A**TP vs. d**C**TP by destabilizing the active site contents of closed pol β and increasing the conformational barrier for thumb subdomain closing in the mismatch.

## Discussion and conclusion

We have described the conformational closing profiles of pol β/DNA/8-oxo**G **complexes with d**C**TP and d**A**TP incoming nucleotides by TPS simulations. Both 8-oxo**G **complexes pass four transition states to reach their closed states: two of the four transitions are the same – thumb subdomain closing (TS1) and Phe272 flip (TS4) – while the others are different – Asp192 flip and Arg258 rotation in the mismatch, and Arg258 half and full rotations in the match.

Importantly, subtle differences in the conformational profiles of the 8-oxo**G **complexes have emerged. First, the matched system must overcome a higher overall free energy barrier than the mismatched complex (18 ± 3 vs. 15 ± 3 k_B_T), but they can be considered similar due to the size of our error bars. The higher overall barrier corresponds to a slightly smaller k_cat _for d**C**TP (2.0 s^-1^) vs. d**A**TP insertion (2.5 s^-1^) opposite 8-oxo**G**.

Second, the free energy barrier for thumb closing in the 8-oxo**G**:d**C**TP complex (~15 k_B_T) is slightly higher than the 8-oxo**G**:d**A**TP complex (~13 k_B_T), and the increased value in the mismatched system arises from repulsive interactions between Tyr271 and the nascent base pair. A probability distribution analysis of the dihedral angle of d**A**TP in the mismatched complex shows that the d**A**TP base is distorted from an *anti *to *syn *conformation as the system traverses transition states 2 to 3 (Asp192 flip and Arg258 rotation), with adenine gradually accommodating itself and returning to the *anti *conformation after the thumb fully closes. In addition, the Tyr271Ala mutant simulation for the mismatched complex indicates that thumb can open quickly after the Tyr271 is replaced with a much less bulky amino acid.

The relative stability of the final closed states compared to the open states emerged as an important factor hampering misincorporation (**G**:**A**) as suggested by prior TPS simulation works. While the energy differences between the closed and open states in the **G**:**C **and **G**:**A **complexes without the lesion is dramatic (-3 vs. 9 k_B_T), here we find a smaller but significant difference, 2 vs. -2 k_B_T, for 8-oxo**G**:**A **and 8-oxo**G**:**C **systems. Significantly, all this information can be now pieced to define a sequence of stability for the closed states of the four complexes relative to their open states: **G**:**C **> 8-oxo**G**:d**C**TP > 8-oxo**G**:d**A**TP > **G**:**A**. This hierarchy directly corresponds to the experimental measurements that indicate both d**C**TP and d**A**TP can be incorporated opposite 8-oxo**G **much more efficiently than an incorrect insertion (**G**:**A**). Particularly, the more stable closed conformation and lower overall conformational energy barrier of pol β associated with 8-oxo**G**:d**A**TP lead to much higher efficiency (62,000-fold increase) for d**A**TP incorporation opposite 8-oxo**G **than d**G**. In addition, the absence of multiple conformational pathways in the 8-oxo**G**:d**A**TP system in contrast to **G**:**A **might also contribute to the observed higher processing efficiency for the former in pol β.

From our stochastic simulations (Fig. 5), we interpret that since the mismatched system spends more time in the open state than the matched system, d**A**TP can dissociate from the ternary complex much easier, and this could lower the binding affinity of d**A**TP to pol β, resulting a large K_m _value and lower efficiency. Indeed, experiments testing the effects of gapped DNA substrates on pol β's fidelity [38] suggest that fidelity is achieved through comparable contributions of the different catalytic constant (k_cat_) and binding affinity (K_m_) in single nucleotide gap-filling synthesis. Thus, our computed data provide insights into the different binding affinities (K_m_) of d**A**TP and d**C**TP in pol β when 8-oxo**G **is the template base.

Unlike the pol β/DNA substrate complex with a **G**:**C **nascent base pair, both matched and mismatched 8-oxo**G **complexes do not have ion-rearrangement step in their closing pathways (Fig. 4). The critical distances for metal-ion binding are larger than the ideal values required for the nucleotidyl-transfer reaction [see Additional file 1] (see additional table 1) in both 8-oxo**G **complexes. These deviations suggest that these systems must go through more subtle rearrangements ("pre-chemistry" avenue) prior to the chemical reaction [39,40]. The existence of additional free energy barriers for the subtle rearrangements is also reflected in the larger catalytic constants (k_cat_) for the matched and mismatched 8-oxo**G **complexes computed using the chemical barrier of a **G**:**C **base pair than their corresponding experimental values (2.5 and 2.0 s^-1 ^vs. 0.6 ± 0.05 and 0.35 ± 0.01 s^-1^, respectively). The additional pre-chemistry checkpoints (energy barriers) following conformational steps might account for the overestimated k_cat _values.

In summary, transition path sampling suggests that, when processing the 8-oxo**G **lesion, pol β differentiates the incoming nucleotides d**C**TP and d**A**TP through altered conformational barriers, closed-state stability, and the requisite active-site geometry before the chemical reaction. Particularly, residue Tyr271 hampers the thumb subdomain closing of the 8-oxo**G**:d**A**TP complex and provides subtle selectivity for pol β to discriminate d**C**TP against d**A**TP.

## Methods

### Systems preparation

We construct open and closed models of the pol β/DNA substrate complexes from the binary (PDB ID code 1BPX) and ternary (PDB ID code 1BPY) crystal structures [41], respectively. Since the binary structure does not contain an incoming nucleotide, a d**C**TP residue and two binding magnesium ions are added to pair with the d**G **template using the ternary crystal structure as reference. The template guanine base in the open and closed pol β complexes is then modified to 8-oxo**G **by adding an oxygen atom on C8 and removing a hydrogen atom from C7. Based on the resulting complexes with 8-oxo**G**:d**C**TP, we create the *syn *conformation for 8-oxo**G **by rotating the guanine base around the N-glycosidic bond (N-C1') by 180° and replace the d**C**TP with a d**A**TP nucleotide using insightII to build the 8-oxo**G **(*syn*):d**A**TP open and closed complexes. All hydrogen atoms are added to the models using CHARMM [42-44].

The 8-oxo**G**:d**A**TP and 8-oxo**G**:d**C**TP model systems are immersed in a face-centered water cube with dimensions of 60.6 Å × 60.6 Å × 60.6 Å using PBCAID [45] and Simulaid [46]. The smallest image distances are set to 16 Å. Overlapping water molecules as well as those within 1.8 Å of the heavy atoms of the protein and DNA are removed. Sodium and chloride ions are added by replacing those solvent water oxygen atoms with the most negative and positive electrostatic potentials (derived using DELPHI [47,48]), respectively, to neutralize the systems and obtain an ionic strength similar to the enzyme's natural environment. All ions are added at least 8 Å away from the heavy atoms of protein and DNA. The four solvated pol β/DNA complexes (open and closed with 8-oxo**G**:d**A**TP; open and closed with 8-oxo**G**:d**C**TP) each contain 335 protein residues, 32 nucleotides, 2 Mg^2+ ^ions, 21 Cl^- ^ions, 43 Na^+ ^ions, and 11830 water molecules, differing only in the incoming nucleotides d**A**TP and d**C**TP.

The pol β/DNA complexes are minimized and equilibrated using CHARMM c28a3 version [42,43] with the CHARMM27 all-atom force fields for nucleic acids and lipids under periodic boundary conditions. The force field parameters for 8-oxo**G **are taken from prior work [39]. For minimization and equilibration, the van der Waals interactions are treated by the switch cutoff method and electrostatic interactions by force-shift method. The nonbonded interaction cutoff distance is set to 12 Å, switch distance to 10 Å, and pair list distance to 14 Å. Bonds to all hydrogen atoms are constrained with SHAKE.

The performance of different long-range truncation methods have been evaluated by Norberg, et al. [49] using a highly charged oligonucleotide in aqueous solution. Results show that the atom-based force-shift method can produce very accurate and stable simulation trajectories and that it is computationally more efficient than the particle mesh Ewald (PME) truncation method.

For each of the four models, the water molecules and all other hydrogen atoms are minimized using the steepest-decent (SD) algorithm for 10,000 steps and an adopted-basis Newton-Raphson (ABNR) minimization for 20,000 steps with all the heavy atoms of protein and DNA fixed. An equilibration of 100 ps is performed at 300 K by Langevin dynamics to relax the sodium and chloride ions around the protein and DNA. Then the system is minimized with all protein and DNA heavy atoms fixed. Finally, the entire system is equilibrated using Langevin dynamics for 100 ps.

After minimization and equilibration, the overall RMSD values (Cα atoms) of the open and closed state models for the 8-oxo**G**:d**A**TP complex relative to their initial structures are 1.09 Å and 1.32 Å, respectively; those for the 8-oxo**G**:d**C**TP complex are 1.12 Å and 1.38 Å, respectively.

### Transition path sampling simulations

The transition path sampling (TPS) method of Chandler and coworkers relies on the idea of importance sampling using standard Monte Carlo (MC) procedures [50-53], which explore sequences of states constituting dynamical trajectories [54,55] through random walks. Namely, starting from an initial trajectory that captures a barrier crossing, TPS samples the trajectory space of a system using Metropolis MC method by performing a random walk with the shooting algorithm [21]; the random walk is biased to make sure that the most important regions of the trajectory space are adequately sampled [55]. The frequency of a trajectory region being visited is determined by its probability so that even when a random walk is initiated far from a representative transition pathway, the bias can drive the system to those important regions of the transition space after sampling. Thus, despite the unphysical nature of the initial sampling trajectories that we obtained using targeted molecular dynamics (TMD), the TPS protocol can lead the system to the most important regions and yield physically meaningful trajectories passing through saddle area.

The shooting algorithm [21] generates an ensemble of new trajectories by perturbing initial momenta of atoms in a randomly chosen time interval while ensuring conservation of Maxwellian distribution of velocities, total linear and angular momentum, and detailed balance. The perturbation scheme employed in our work is also symmetric – the probability of generating a new set of momenta from the old set is the same as the reverse probability of generating the old set from the new set.

In particular, the ensemble of new trajectories {χ_τ_} of length τ are generated by a Metropolis algorithm according to a path action S{χ}_τ_: S{χ}_τ _= ρ(0) h_A_(χ_0_)H_B_{χ}_τ_, where ρ(0) is the probability of observing the configuration at t = 0 (ρ(0) ∝ exp(-βE(0)) in the canonical ensemble). The newly generated trajectories are accepted or rejected based on selected statistical criteria that characterize the ensemble of trajectories [34,55].

The ergodicity and convergence of each TPS run is confirmed by calculating the autocorrelation function of the order parameter ⟨χ_*i*_(0)χ_*i*_(τ)⟩ associated with each transition state i, where ⟨·⟩ denotes the average over the ensemble of generated trajectories. For each transition state, the autocorrelation function is plotted from ⟨χ_*i*_(0)χ_*i*_(0)⟩ ≈ ⟨χ_*A*_⟩^2 ^to the time τ where ⟨χ_*i*_(0)χ_*i*_(τ)⟩ ≈ ⟨χ_*A*_⟩⟨χ_*B*_⟩; this range is spanned during our sampling time τ [see Additional file 1] (see additional figures 5 and 6), indicating that the transition state regions between A and B are crossed during this interval. The time used for the gradual transition of the autocorrelation function ⟨χ_*i*_(0)χ_*i*_(τ)⟩ from these plots can provide an estimate for the timescale of barrier crossing (τ_mol_). Thus, the length of the MD trajectories should be longer than the τ_mol _value to sufficiently cover the entire transition region.

The above procedure both conserves the equilibrium distribution of individual metastable states and ensures that the accepted molecular dynamics trajectories connect the two metastable states for a particular transition. The shooting algorithm used in our work based on the Metropolis scheme [56] also conserves microscopic reversibility. Hence, the ensemble of MD trajectories generated is guaranteed to converge to the correct ensemble defined by the path action and represents configurations that constitute the correct pathway for hopping between the metastable states.

The detailed application of transition path sampling (TPS) to characterize the transition regions of a complex requires the knowledge of an initial trajectory connecting the stable starting and final states of the system and order parameters that can efficiently define the two states. To obtain the initial trajectories, we apply targeted molecular dynamics (TMD) simulations to connect the open and closed states of the pol β complexes with 8-oxo**G**:d**A**TP and 8-oxo**G**:d**C**TP base pairs. To choose appropriate order parameters for TPS simulations, we use the crystallographic data [41], molecular dynamics [57,58], and prior TPS [31,32] studies on pol β as reference. Since these works have shown that key active-site residues (Asp192, Arg258, and Phe272) and α-helix N on the thumb subdomain serve as measures of pol β's closing pathway, we start testing values of dihedral angles associated with these residues and the RMSD value of α-helix N and then identify the proper order parameters for the two pol β 8-oxo**G **complexes.

The TMD code implemented in CHARMM c28a2 [43] is used to generate the initial constrained trajectories. An energy restraint based on the RMS distance of the system relative to the final state is applied to force the open pol β complexes to close. The restraint energy can be expressed by:

*E*_*RMS *_= *K*[*D*_*RMS*_(*X*(*t*),*X*^target^) - *d*_0_]^2^

In this equation, K is a force constant, D_RMS _is the relative RMS distance for a selected set of atoms between the instantaneous conformation X(t) and the reference X^target^, and d_0 _is an offset constant (in Å). In our TMD simulations, the RMS distance is evaluated using the heavy atoms on α-helix N of pol β's thumb subdomain. A total force constant of 2000 kcal mol^-1 ^Å ^-2 ^is applied to all heavy atoms of the open complex except those of residues 1–9 because they are missing in the ternary crystal structure. The offset parameter d_0 _is set to decrease from 3.2 Å to 0 by 0.16 Å every 2 ps.

With d_0 _decreasing as a function of simulation time, pol β is thus driven from the open to the closed conformation.

From the TMD trajectories of pol β 8-oxo**G **complexes linking their open and closed states, we extract the time evolution of dihedral angles of the key residues (Asp192, Arg258, and Phe272). For the thumb subdomain closing motion, we analyze the RMSD of α-helix N relative to the closed conformation along the TMD trajectory. The time-evolution plots for the residues and thumb subdomain conformational changes in the two complexes are shown by [Additional file 1] (see additional figures 1 and 2), where clear transitions of the dihedral angles and RMSD are observed. The transition region for each conformational change is thus identified from these plots.

Since the TMD trajectories for the two 8-oxo**G **systems are constrained and nonphysical, we select frames that locate on the transition regions of the residues and thumb conformational changes and perform unconstrained dynamics simulations. We perturb the atomic momenta of the frames and integrate the equations of motion forward and backward for a relatively short time to generate new physical, unbiased trajectories that connect the open and closed states of pol β. Based on these unconstrained simulations, we determine the adequate length of sampling trajectories for all the transition states. Specifically, for the 8-oxo**G**:d**A**TP complex, the trajectories for Asp192 flip and Arg258 rotation are simulated for 10 ps, and those for Phe272 flip are run for 20 ps. For the 8-oxo**G**:d**C**TP complex, the sampling paths for Asp192 flip and Arg258 half-rotation are 10 ps in length, and those for Phe272 flip are 20 ps. To capture the transition states of thumb closing in the two complexes, the sampling trajectories have to be propagated for 100 ps.

Using one of the newly generated physical trajectory as the starting trajectory, we perform path sampling for each individual conformational change with the shooting and shifting algorithm [33] and a Monte Carlo protocol [34]. The entire process is implemented in a PERL script by calling CHARMM to generate new trajectories.

For the shooting move [34], we add a random perturbation (momentum displacement) drawn from a Gaussian distribution to the old momentum vector of the system. The total linear momentum of the new momentum is set to zero by subtracting δ_i_p_i_'/N from all single particle momenta. Next, the total angular momentum is set to zero. This is done in the CHARMM program by setting NTRFRQ ≠ 0. Then the new momenta of the system are calculated and a new trajectory is generated by running molecular dynamics simulations backward and forward. The new path is accepted or rejected into the transition path ensemble according to a Metropolis criterion. In a shifting move [34], a segment of a trajectory is deleted from either the beginning or the end of an existing path connecting the open and closed states. A new trajectory segment of the same length as the deleted one is regenerated from the opposite end of the original trajectory, so that the new path is still of the same total length. The shifting move is especially useful when the shooting algorithm is trapped in an area without generating newly accepted trajectories.

As recommended by Dellago et al. [59], to ensure sufficient sampling an acceptance rate of 30 to 60% is obtained for the path sampling of each transition state by varying the momentum perturbation magnitude from 0.0005 to 0.001. A total of 100 accepted trajectories are collected to map each transition state.

The convergence of the harvested sampling trajectories is verified by computing the autocorrelation function associated with order parameters to check for decorrelation of paths. Results of the autocorrelation functions are included in [Additional file 1] (see additional figures 5 and 6). The new trajectories are essentially decorrelated if the autocorrelation function shows a gradual transition between ⟨χ_A_⟩^2 ^and ⟨χ_A_⟩⟨χ_B_⟩.

### Free energy barrier and rate constant calculations

The free energy barriers for transition states are evaluated using the "BOLAS" protocol described in [34]. Namely, we divide the reaction coordinate of each transition into 10 small overlapping windows and perform umbrella sampling to generate 100 trajectories on each window. We then combine the potential of mean force plots obtained from the sampling calculations on each window by adding/subtracting a constant to match the free energy values of the overlapping region. From the overall free energy plots, we calculate the free energy barriers for the conformational transitions. The error bar for the free energy calculations is determined by repeating umbrella sampling on one window of a transition for five times with the same initial trajectory but different starting pseudorandom numbers. The largest standard deviation (~3 k_B_T) is used as the error bar.

The rate of the transition between adjoining metastable states is estimated using transition state theory [60]as:

kTSTAB=1τmole−βΔFABbarrier
 MathType@MTEF@5@5@+=feaafiart1ev1aaatCvAUfKttLearuWrP9MDH5MBPbIqV92AaeXatLxBI9gBaebbnrfifHhDYfgasaacH8akY=wiFfYdH8Gipec8Eeeu0xXdbba9frFj0=OqFfea0dXdd9vqai=hGuQ8kuc9pgc9s8qqaq=dirpe0xb9q8qiLsFr0=vr0=vr0dc8meaabaqaciaacaGaaeqabaqabeGadaaakeaacqWGRbWAdaqhaaWcbaGaemivaqLaem4uamLaemivaqfabaGaemyqaeKaemOqaieaaOGaeyypa0ZaaSaaaeaacqaIXaqmaeaaiiGacqWFepaDdaWgaaWcbaGaemyBa0Maem4Ba8MaemiBaWgabeaaaaGccqWGLbqzdaahaaWcbeqaaiabgkHiTiab=j7aIjabfs5aejabdAeagnaaDaaameaacqWGbbqqcqWGcbGqaeaacqWGIbGycqWGHbqycqWGYbGCcqWGYbGCcqWGPbqAcqWGLbqzcqWGYbGCaaaaaaaa@4E75@

### MD simulation of the Y271A mutant intermediate complex with 8-OxoG:dATP

The equilibrated wild-type intermediate complex of pol β/DNA with 8-oxo**G **(*syn*):d**A**TP (*anti*) before chemistry is obtained from the prior MD work [20]. Residue Tyr271 on pol β is replaced by an alanine using InsightII [61] to construct the Y271A mutant. The distortions introduced by the replacement are removed by constrained minimization of the complex with all residues other than 271 frozen. The entire complex is then minimized using the SD and ABNR methods (convergence criterion is that the gradient of root-mean-square deviation is less than 10^-6 ^kcal/mol Å). The complex is equilibrated for 300 ps before starting the production simulation for 14.2 ns using the LN integrator [62-65]. The RMSD of Cα atoms of the equilibrated complex relative to the initial model is 0.86 Å.

## Abbreviations

Pol β, DNA polymerase β; 8-oxo**G**, 8-oxoguanine; d**C**TP, 2'-deoxyribocytidine 5'-triphosphate; d**A**TP, 2'-dideoxyriboadenosine 5'-triphosphate; TPS, transition path sampling; TMD, targeted molecular dynamics

## Competing interests

The author(s) declare that they have no competing interests.

## Authors' contributions

YW perceived and designed the work. YW performed the computational study and drafted the manuscript. YW and TS interpreted data and revised the manuscript.

## Supplementary Material

Additional file 1 Analyses of the conformational and energetic properties of the pol β/DNA complexes with 8-oxo**G**. The data provided detail information collected from targeted molecular dynamics, transition path sampling, umbrella sampling, and molecular dynamics simulations as well as sampling convergence and method reliability tests. Click here for file

## Figures and Tables

**Table 1:** Transition states properties for the closing conformational profiles of the pol β 8-oxo**G **complexes with d**C**TP and d**A**TP incoming nucleotides.

Pol β/DNA/8-oxo**G **complexes	Transition states	Order parameters^a^	τ^b ^(ps)	ΔE_AB _vs ΔE_BA_^c^	Rate constants^d^
Binding d**A**TP	Thumb closing	RMSD of α-helix N (4.5 Å to 2.5 Å)	60	13 4	32000 2.6 × 10^8^
	Asp192 flip	C-Cα-Cβ-Cγ (80° to 150°)	9.6	6 7	3.0 × 10^8^ 9.5 × 10^7^
	Arg258 rotation	Cβ-Cγ-Cδ-Nε (100° to 150°)	5.2	10 16	2.4 × 10^7^ 1.6 × 10^5^
	Phe272 flip	Cα-Cβ-Cγ-Cδ1 (-70° to 40°)	4.0	3 8	4.6 × 10^4^ 8 × 10^8^
	Nucleotidyl transfer		0.2		9.4
Binding d**C**TP	Thumb closing	RMSD of α-helix N (4.5 Å to 2.5 Å)	55	15 5	7200 9.6 × 10^7^
	Arg258 half-rotation	Cβ-Cγ-Cδ-Nε (-95° to -150°)	3	5 7	2.2 × 10^9^ 3.0 × 10^8^
	Arg258 full-rotation	Cγ-Cδ-Nε-Cζ (100° to 150°)	7.9	7 10	4.2 × 10^7^ 3.8 × 10^6^
	Phe272 flip	Cα-Cβ-Cγ-Cδ1 (-70° to -150°)	6.0	4 7	1.2 × 10^10^ 1.5 × 10^8^
	Nucleotidyl transfer		0.2		9.4

a. Order parameters for transition states on the conformational closing pathways of the two complexes. The transition regions are shown in parentheses.

b. Time required to transverse the transition region as obtained from the correlation function of each transition path sampling [see Additional file 1] (see additional figures 5 and 6).

c. Free energy barriers ΔE_AB _and ΔE_BA_computed by performing umbrella sampling using the "BOLAS" program [34]. Detailed free energy plots for each transition are shown in [Additional file 1] (see additional figures 7 and 8).

d. Rate constants for each transition estimated from equation

kTSTAB=1τmole−βΔFABbarrier
 MathType@MTEF@5@5@+=feaafiart1ev1aaatCvAUfKttLearuWrP9MDH5MBPbIqV92AaeXatLxBI9gBaebbnrfifHhDYfgasaacH8akY=wiFfYdH8Gipec8Eeeu0xXdbba9frFj0=OqFfea0dXdd9vqai=hGuQ8kuc9pgc9s8qqaq=dirpe0xb9q8qiLsFr0=vr0=vr0dc8meaabaqaciaacaGaaeqabaqabeGadaaakeaacqWGRbWAdaqhaaWcbaGaemivaqLaem4uamLaemivaqfabaGaemyqaeKaemOqaieaaOGaeyypa0ZaaSaaaeaacqaIXaqmaeaaiiGacqWFepaDdaWgaaWcbaGaemyBa0Maem4Ba8MaemiBaWgabeaaaaGccqWGLbqzdaahaaWcbeqaaiabgkHiTiab=j7aIjabfs5aejabdAeagnaaDaaameaacqWGbbqqcqWGcbGqaeaacqWGIbGycqWGHbqycqWGYbGCcqWGYbGCcqWGPbqAcqWGLbqzcqWGYbGCaaaaaaaa@4E75@

where τ is the time to transverse the energy barrier, and ΔF is the free energy barrier value.
