# Supplementary material for: Distinct energetics and closing pathways for DNA polymerase β with 8-oxoG template and different incoming nucleotides
Source: BMC Struct Biol. 2007 Feb 21;7:7. doi: 10.1186/1472-6807-7-7 (PMC1819382; doi:10.1186/1472-6807-7-7)
Supplement: Additional file 1 — Analyses of the conformational and energetic properties of the pol β/DNA complexes with 8-oxoG. The data provided detail information collected from targeted molecular dynamics, transition path sampling, umbrella sampling, and molecular dynamics simulations as well as sampling convergence and method reliability tests. [file 1472-6807-7-7-S1.pdf]

Distinct energetics and closing pathways for DNA polymerase β with 8-oxoG template and different incoming nucleotides

Yanli Wang and Tamar Schlick*

Department of Chemistry and Courant Institute of Mathematical Sciences, New York University, 251 Mercer Street, New York, New York 10012, USA

Additional data

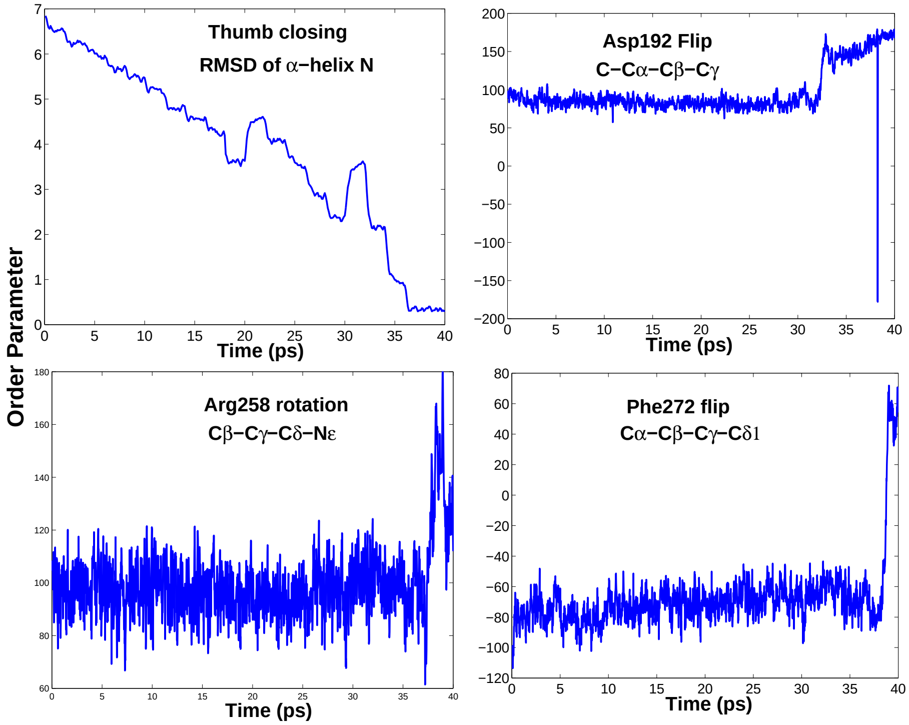

Figure 1: Initial constrained trajectories for the conformational transitions in the 8-oxo**G**:d**A**TP complex obtained from TMD.

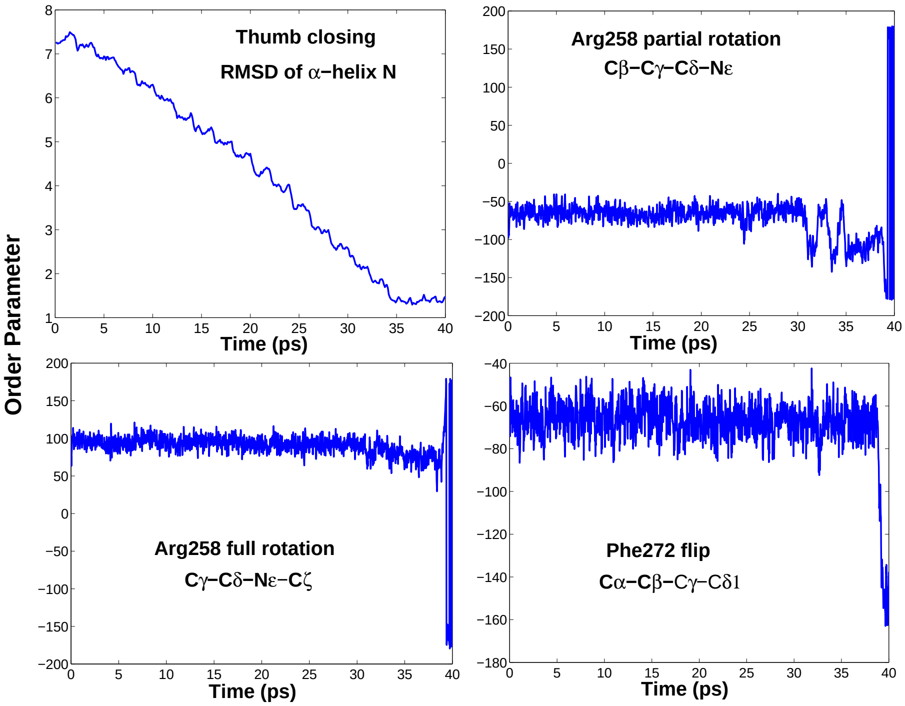

Figure 2: Initial constrained trajectories for the conformational transitions in the 8-oxo**G**:d**C**TP complex obtained from TMD.

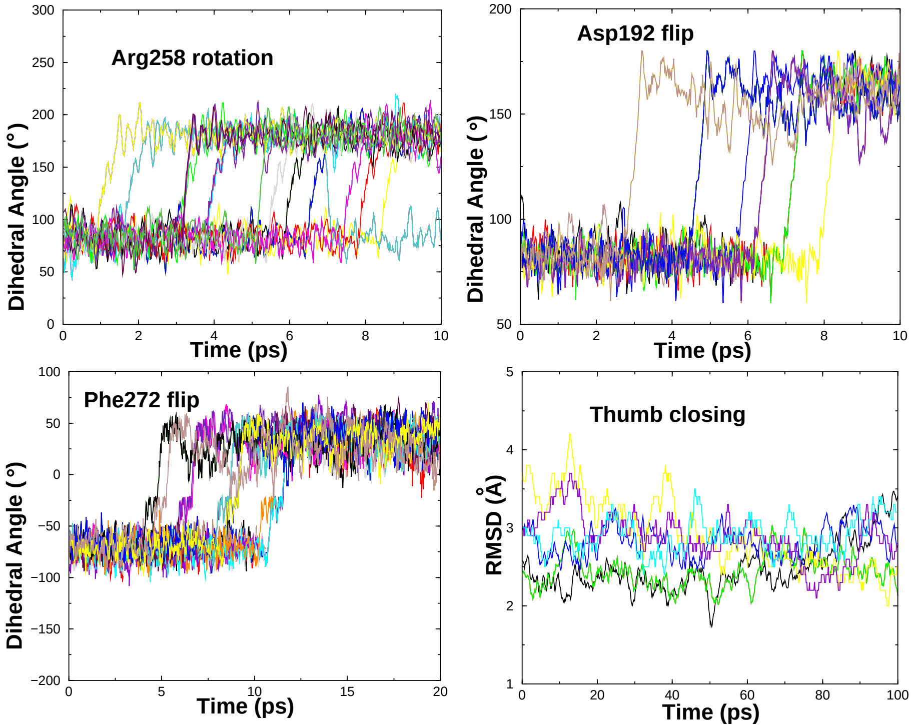

Figure 3: Sample unconstrained trajectories for the transition states in the closing profile of the 8-oxo**G**:d**A**TP complex.

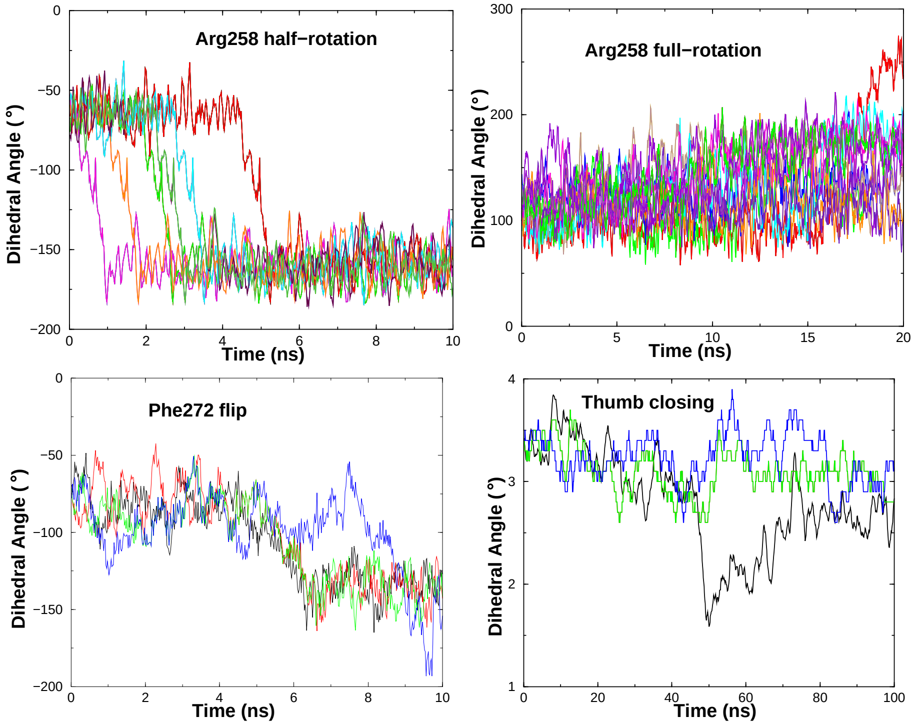

Figure 4: Sample unconstrained trajectories for the transition states in the closing profile of the 8-oxo**G**:d**C**TP complex.

Test for convergence of path sampling

To verify the convergences of the harvested trajectories in TPS simulations of the transition states, we computed the time correlation function for each path sampling simulation $\langle\chi(0)\chi(t)\rangle$, where $\chi(0)$ is the order parameter value at time 0, i.e. χ_A, $\chi(t)$ is the order parameter value at simulation time t, and $\langle\cdot\rangle$ denotes the ensemble average over all the accepted paths of a transition state. At the end of each accepted trajectory, $\chi(t)$ is expected to reach the target basin of the transition, that is, χ_B.

Additional Figs. 5 and 6 show the time correlation functions for all the transition states in the 8-oxo**G**:d**A**TP and 8-oxo**G**:dCTP complexes, respectively. These plots indicate convergence to the final state B in all the accepted trajectories.

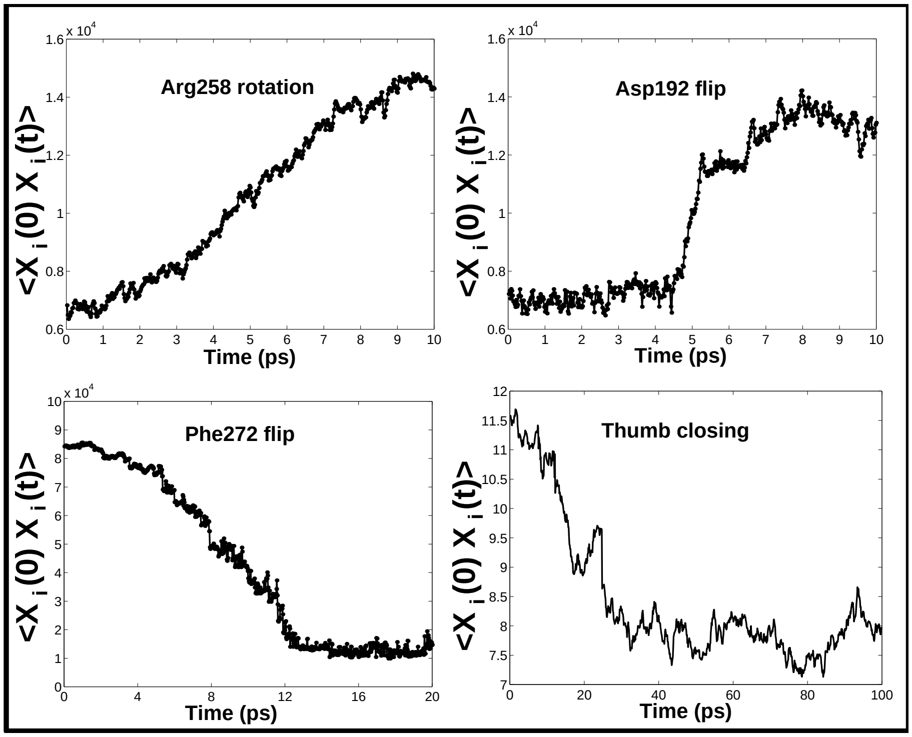

Figure 5: Correlation functions of order parameters for transiton states in the closing pathway of the 8-oxo**G**:d**A**TP complex.

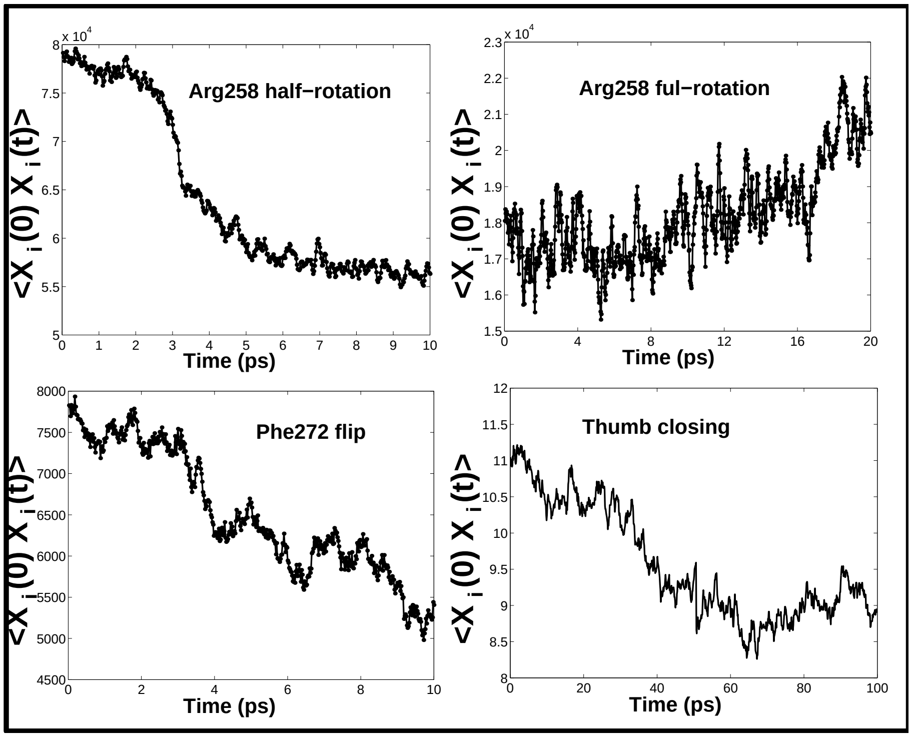

Figure 6: Correlation functions of order parameters for transition states in the closing pathway of the 8-oxo**G**:d**C**TP complex.

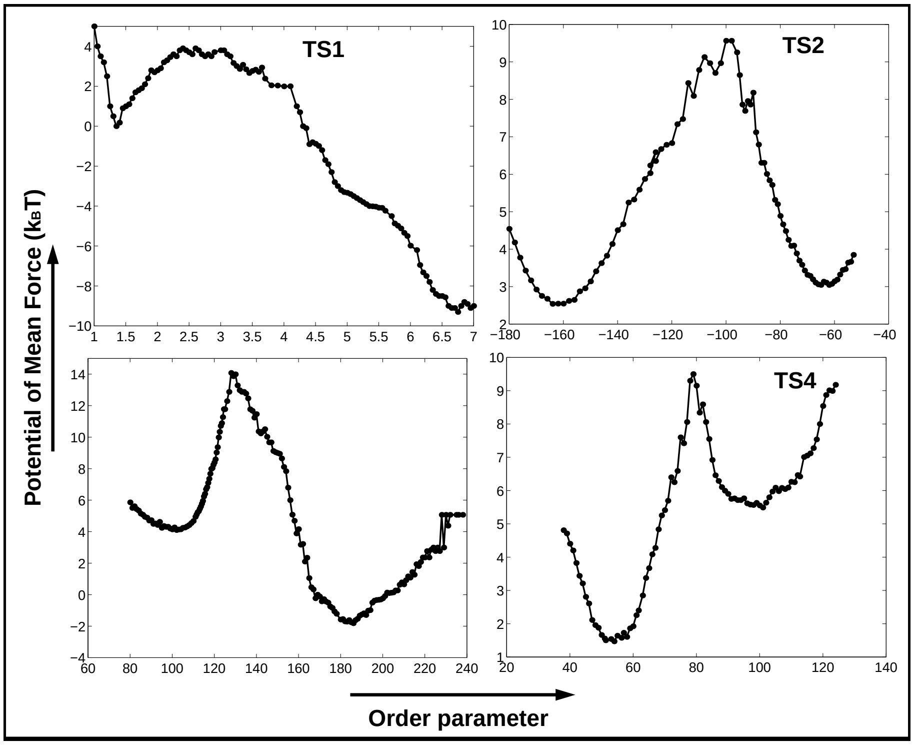

Figure 7: Potential of mean force plots for the 8-oxo**G**:d**C**TP complex computed by umbrella sampling.

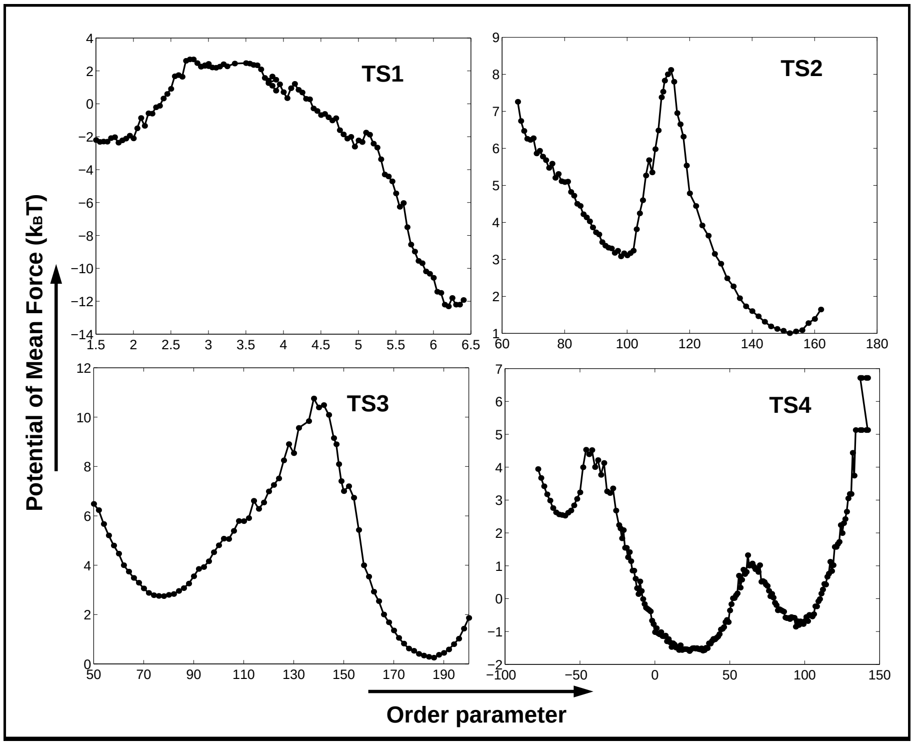

Figure 8: Potential of mean force plots for the 8-oxo**G**:d**A**TP complex computed by umbrella sampling.

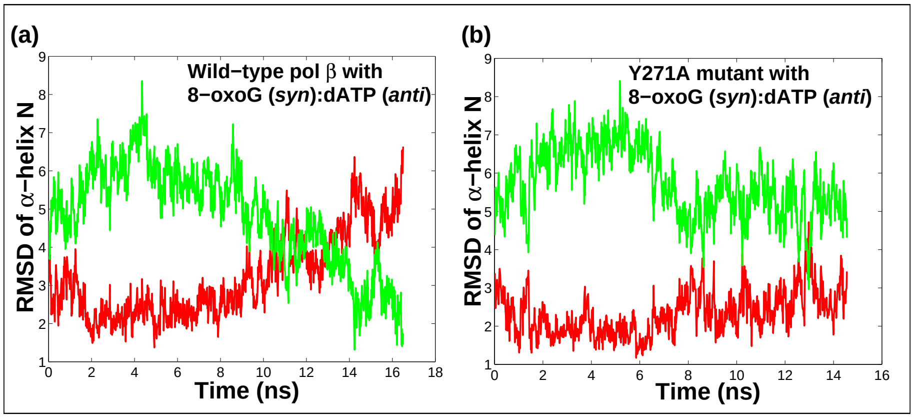

Figure 9: The RMSD evolution of α-helices N on the wild-type (a) and mutant (b) complexes relative to the open (green) and closed (red) crystal structures.

Table 1.The average critical distances for the chemical reaction after the final transitions of pol β/DNA complexes with 8−oxoG (*syn*):dATP (*anti*) and 8−oxoG (*anti*):dCTP(*anti*) compared to those of the crystallographic data.

Critical distances	8−oxoG:dATP	8−oxoG:dCTP	Crystal data*
O3′−Pα	5.49	6.65	3.40
Mg2−O1α	4.42	6.40	2.19
Mg2−O3′	6.96	2.04	2.19
Mg2−OD1−Asp190	1.92	1.89	3.37
Mg2−OD2−Asp190	1.89	1.87	2.05
Mg2−OD1−Asp192	1.90	1.80	2.01
Mg2−OD2−Asp192	2.17	3.09	3.33
Mg2−OD2−Asp256	1.83	1.79	2.15
Mg1−OD2−Asp192	3.95	5.36	2.02
Mg1−OD1−Asp190	5.03	5.96	1.95
Mg1−O−Asp190	2.11	4.80	4.12
Mg1−O1α−ATP	1.86	1.85	2.06
Mg1−O2β−ATP	1.90	3.16	2.04
Mg1−O1γ−ATP	1.80	1.83	2.08

*** The PDB ID code for the crystal structure is 2FMS.**

Testing the TPS method on a model system — sugar puckering in dG

To validate the reliability of our TPS method, we reproduced the conformational transition pathway of sugar puckering between the C2′-endo and C3′-endo of d**G** and the associated free energy barrier using the protocol employed in our study for pol β complexes with 8-oxo**G**.

The pseudorotation angle (p) of a nucleoside defines the sugar conformation based on a wave-like motion from chosen mean plane defined by five ring atoms [1]. The definition of the pseudorotation angle (p) can be found in [2–4]. The sugar puckering conformational space for standard DNA is characterized by two energy minima at C3′-endo and C2′-endo conformations, also called north and south ranges, centering around p=18° and p=162°, respectively. From experimental structures of DNA duplexes, typical C3′-endo state lies in the N (north) range of pseudorotational values, -1° $\leq$ p $\leq$ 34°, and the C2′-endo is in the S (south) range, 137° $\leq$ p $\leq$ 194°. The interconversion between C2′-endo and C3′-endo deoxyribose conformations has been investigated by many theoretical studies [4–14]. The estimated energy barrier value for deoxyribose sugar puckering with the sugar in its ambient solvent environment is about 1.5 to 5 kcal/mol according to [4,6,9,14].

For our study, we use the published Cartesian coordinates (see Additional Fig. 10) for the d**G** in C2′-endo and C3′-endo energy minima computed at MP2/6-31G* level of theory [10]. The two d**G** molecules are then solvated in a water box with a dimension of 20.04 Å × 22.40 Å × 26.27 Å containing 329 water molecules. The resulting models

are minimized and equilibrated following the procedure described in the Method section of the main text. An initial tentative trajectory linking the C2′-endo and C3′-endo conformations is generated using TMD. TPS simulations are performed to sample the conformational space for sugar puckering with each trajectory of 20 ps long. 100 such trajectories are harvested to map the conformational pathway. The free energy barrier for the C2′-endo and C3′-endo interconversion is computed by umbrella sampling ("BOLAS" [14]) simulations. The length of the umbrella sampling trajectory is set to 10 ps and 150 trajectories are collected for each of the 10 sampling windows.

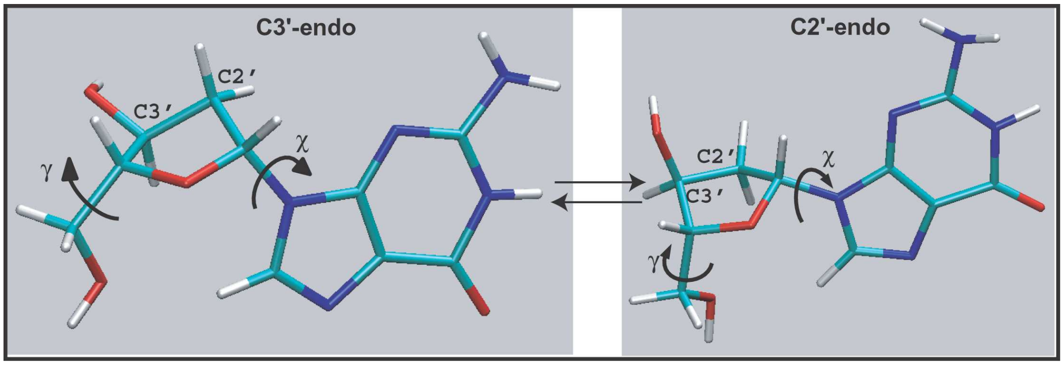

Figure 10: The C3′-endo (left) and C2′-endo (right) sugar puckering conformations of d**G**.

As seen from the computed two-dimensional distribution plot of the pseudorotation angle (p) and the phase amplitude (Additional Fig. 11), the pseudorotation angle p is mainly clustered around two regions, 0-30° (C3′-endo) and 100-180° (C2′-endo), which represent the two metastable states. The barrier region separating them is the bottleneck for the transition.

Additional Fig. 12 shows the final energetic profile of d**G** sugar puckering resolved

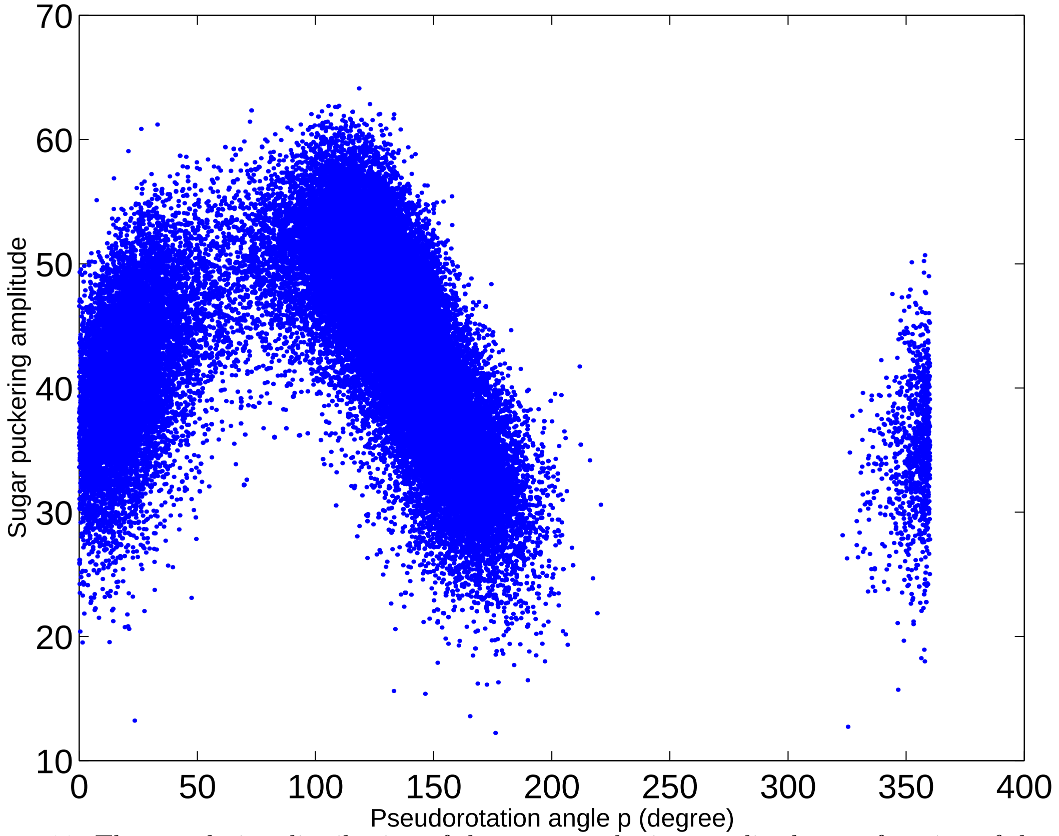

Figure 11: The population distribution of the sugar puckering amplitude as a function of the pseudorotation phase angle (p) for all structures observed in 1500 TPS trajectories connecting the d**G** C2′-endo and C3′-endo conformations.

from umbrella sampling: the C2′-endo conformation appears to be thermodynamically favored over the C3′-endo conformation by 0.37 kcal/mol; the free energy barrier for the transition between C2′-endo and C3′-endo is 1.92 kcal/mol. These free energy values are in good agreement with previous experimental and theoretical estimates on nucleosides [4–6,9,12–16].

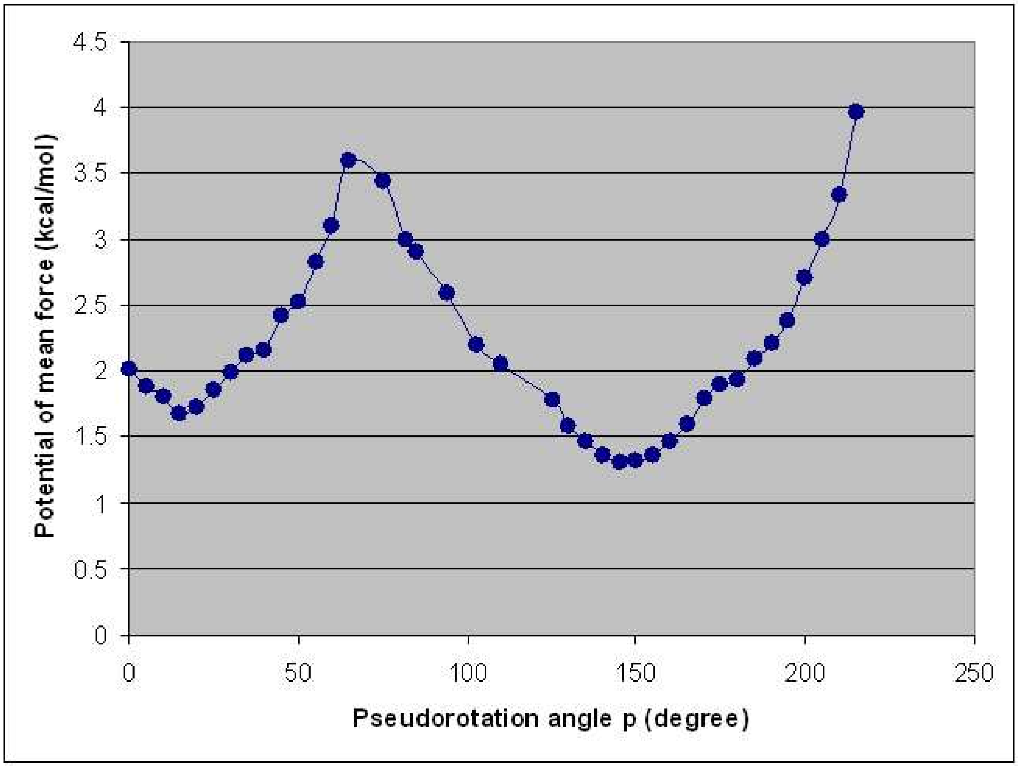

Figure 12: The potential of mean force plot for sugar puckering interconversion between C2′-endo and C3′-endo of d**G** calculated by umbrella sampling simulations.

References

1. T. Schlick. *Molecular Modeling and Simulation: An Interdisciplinary Guide.* Springer-Verlag, New York, NY, 2002.

2. S. Rao, E. Westhof, and M. Sundralingam. Exact method for the calculation of pseudorotation parameters p, m and their errors. a comparison of the altona-sundaralingam and cremer-pople treatment of puckering of five-membered rings. *Acta Crystallogr. A*, 37:421–425, 1981.

3. C. Altona and M. Sundaralingam. Conformational analysis of the sugar ring in nucleosides and nucleotides. A new description using the concept of pseudorotation. *J. Amer. Chem. Soc.*, 94:8205–8212, 1972.

4. K. Arora and T. Schlick. Deoxyadenosine sugar puckering pathway simulated by the stochastic difference equation algorithm. *Chem. Phys. Lett.*, 378:1–8, 2003.

5. M. Levitt and A. Warshel. Extreme conformational flexibility of the furanose ring in DNA and RNA. *J. Amer. Chem. Soc.*, 100:2607–2613, 1978.

6. S. C. Harvey and M. Prabhakaran. Ribose puckering: Structure, dynamics, energetics, and the pseudorotation cycle. *J. Amer. Chem. Soc.*, 108:6128–6136, 1986.

7. H. Gabb and S. C. Harvey. Conformational transitions in potential and free energy space for furanoses and 2′-deoxynucleosides. *J. Amer. Chem. Soc.*, 115:4218–4227, 1993.

8. W. K. Olson and P. J. Flory. Spatial configuration of polynucleotide chains. ii. Conformational energies and the average dimensions of polyribonucleotides. *Biopolymers*, 11:25–, 1972.

9. W. K. Olson. How flexible is the furanose ring? 2. An updated potential energy estimate. *J. Amer. Chem. Soc.*, 104:278–286, 1982.

10. N. Foloppe and J. A. D. Mackerell. Conformational properties of the deoxyribose and ribose moieties of nucleic acids: A quantum mechanical study. *J. Phys. Chem. B*, 102:6669–6678, 1998.

11. T. Schlick, C. Peskin, S. Broyde, and M. Overton. An analysis of the structural and energetic properties of deoxyribose by potential energy methods. *J. Comput. Chem.*, 8:1199–1224, 1987.

12. L. Nilsson and M. Karplus. Empirical energy functions for energy minimization and dynamics of nucleic acids. *J. Comput. Chem.*, 7:591–616, 1986.

13. W. D. Cornell, P. Cieplak, C. I. Bayly, I. R. Gould, J. K. M. Merz, M. D. Feruguson, D. C. Spellmeyer, T. Fox, J. W. Caldwell, and P. A. Kollman. A second generation force field for the simulation of proteins, nucleic acids, and organic molecules. *J. Amer. Chem. Soc.*, 117:5179–5197, 1995.

14. R. Radhakrishnan and T. Schlick. Biomolecular free energy profiles by a shooting/umbrella sampling protocol, "BOLAS". *J. Chem. Phys.*, 121:2436–2444, 2004.

15. J. Wang, P. Cieplak, and P. A. Kollman. How well does a restrained electrostatic potential (resp) model perform in calculating conformational energies of organic and biological molecules? *J. Comput. Chem.*, 21:1049–1074, 2000.

16. D. R. Davis. Conformations of nucleosides and nucleotides. *Prog. Nucl. Magn. Reson. Spectrosc.*, 12:135–225, 1978.
